# Impact of Heart Rate on Myocardial Salvage in Timely Reperfused Patients with ST-Segment Elevation Myocardial Infarction: New Insights from Cardiovascular Magnetic Resonance

**DOI:** 10.1371/journal.pone.0145495

**Published:** 2015-12-30

**Authors:** Luca Arcari, Sara Cimino, Laura De Luca, Marco Francone, Nicola Galea, Manuela Reali, Iacopo Carbone, Carlo Iacoboni, Luciano Agati

**Affiliations:** 1 Department of Cardiology, Sapienza University of Rome, Rome, Italy; 2 Department of Radiology, Sapienza University of Rome, Rome Italy; Indiana University School of Medicine, UNITED STATES

## Abstract

**Background:**

Previous studies evaluating the progression of the necrotic wave in relation to heart rate were carried out only in animal models of ST-elevated myocardial infarction (STEMI). Aim of the study was to investigate changes of myocardial salvage in relation to different heart rates at hospital admission in timely reperfused patients with STEMI by using cardiovascular magnetic resonance (CMR).

**Methods:**

One hundred-eighty-seven patients with STEMI successfully and timely treated with primary coronary angioplasty underwent CMR five days after hospital admission. According to the heart rate at presentation, patients were subcategorized into 5 quintiles: <55 bpm (group I, n = 44), 55–64 bpm (group II, n = 35), 65–74 bpm (group III, n = 35), 75–84 bpm (group IV, n = 37), ≥85 bpm (group V, n = 36). Area at risk, infarct size, microvascular obstruction (MVO) and myocardium salvaged index (MSI) were assessed by CMR using standard sequences.

**Results:**

Lower heart rates at presentation were associated with a bigger amount of myocardial salvage after reperfusion. MSI progressively decreased as the heart rates increased (0.54 group I, 0.46 group II, 0.38 group III, 0.34 group IV, 0.32 group V, p<0.001). Stepwise multivariable analysis showed heart rate, peak troponin and the presence of MVO were independent predictor of myocardial salvage. No changes related to heart rate were observed in relation to area at risk and infarct size.

**Conclusions:**

High heart rates registered before performing coronary angioplasty in timely reperfused patients with STEMI are associated with a reduction in salvaged myocardium. In particular, salvaged myocardium significantly reduced when heart rate at presentation is ≥85 bpm.

## Introduction

In patients with ST-elevated myocardial infarction (STEMI), timely reperfusion is able to preserve part of the area at risk (AAR) from necrosis granting an amount of myocardial salvage resulting from the difference between AAR and final infarct size (IS). Myocardial salvage can be also modified by different factors, since for a same time of occlusion the quantity of myocardial salvage can be different. It is important to better understand the determinants of progression of necrotic wave [1] to develop new reperfusion strategies able to maximize the salvaged area and improve clinical parameters and prognosis [2, 3]. In last year's, several studies have been carried out in animals demonstrating that higher heart rates during the acute phase of STEMI were associated with larger myocardial damage, regardless of the time of coronary occlusion [4,5,6]. Nevertheless, still there is a lack of evidence in humans, since for a long time, histological examination has been the only existing technique to quantify the amount of salvaged myocardium. Recently cardiac magnetic resonance (CMR) has been developed as a well validated and reproducible technique allowing quantification of AAR, IS and myocardial salvage in vivo [2, 3, 7, 8]. Myocardial salvage can be assessed in humans by comparing T2-weighted (edematous myocardium) and late gadolinium enhancement (LGE) CMR images [2, 3, 7, 8]. The aim of this study was to investigate the impact of heart rate, measured before the recanalization, on myocardial damage assessed by CMR in patients with STEMI timely reperfused by primary percutaneous coronary intervention (PPCI).

## Methods

### Study Population

One-hundred eighty seven consecutive patients with first STEMI undergoing PPCI within 6 hours after the onset of symptoms were prospectively enrolled in the study between January 2014 and February 2015. Heart rate was registered in the emergency room before any drug administration and before reperfusion by a caliper on the diagnostic electrocardiogram. Troponin I measurement was also systematically performed at hospital admission, every 3 h for the subsequent 24 h, and then every 12 h for the following 2 days. The CMR study was carried out on day 5 after PPCI. Exclusion criteria were: acute administration of beta-blockers before emergency room admission, atrial fibrillation, unsuccessful PPCI, rescue PCI, facilitated PCI, Killip class III-IV, previous myocardial infarction, previous coronary artery bypass grafting, and contraindications to CMR. Patients with hemodynamic instability at the time of CMR also were excluded. All participants gave written informed consent to the protocol, and the study was approved by the ethical committee of the Department of Cardiology, Policlinico Umberto I, Roma Italy.

### Coronary Angioplasty

PPCI and stenting of infarct related artery was performed in all patients according to the clinical protocol used at our institution [9,10]. Thrombolysis in Myocardial Infarction (TIMI) flow grade was semi quantitatively scored as previously described [11]. The number of coronary vessels demonstrating significant coronary artery disease was reported. A successful angioplasty was defined a combination of post-procedural TIMI flow grade 3 and residual stenosis <30%. Time to reperfusion was defined as the interval from the onset of symptoms to the first balloon inflation. The grade of epicardial collaterals to the infarcted-related artery was evaluated according to Rentrop et al.[12].

### CMR Acquisition Protocol

CMR studies were performed with a 1.5-T unit Avanto Siemens, Erlangen, Germany. All studies were performed with the use of dedicated cardiac software, phased array surface receiver coil, and ECG triggering. In brief, after determination of cardiac axes with localizers, breath-hold steady state free-precession cine CMR was performed in cardiac vertical and horizontal long-axis orientation and in short-axis orientation. In cardiac short-axis orientation, both ventricles were completely encompassed by a stack of contiguous slices. Next, myocardial edema imaging was performed with the use of breath-hold black-blood T2-weighted, short-inversion time, inversion-recovery, fast spin-echo imaging (T2w imaging) in cardiac short-axis orientation. Finally, a breath-hold T1- weighted, 2-dimensional contrast-enhanced, inversion recovery, segmented gradient-echo sequence was used to depict the presence, location, and extent of IS (LGE imaging) and the concomitant presence of microvascular obstruction. An intravenous contrast agent dose of 0.1 mmol/kg gadolinium-BOPTA (Multihance, Bracco, Milan, Italy) was used. LGE imaging was performed between 10 and 20 minutes after contrast administration. Inversion time was individually adapted to nullify the signal of remote myocardium (usual range, 220 to 300 ms). Technical details of these sequences are described elsewhere [2, 13, 14]

### Image Analysis

All CMR studies were analyzed off-line by the use of a dedicated workstation (Siemens Argus, Erlangen, Germany) by two experts (MF and IC) who had no knowledge of the patient identity. Conflicts in data interpretation were resolved by consensus. Left ventricular volumes, systolic function, and mass were calculated from the short-axis steady-state free precession cines. Infarcted myocardial mass and microvascular obstruction (MVO) were manually traced and calculated from the LGE short-axis images. As reported in Bondarenko et al. [15], myocardial regions was considered infarcted if the IS signal intensity was >5 standard deviations above the remote myocardium. The MVO was defined as a dark zone within the infarcted segments, usually located in the subendocardium. The mass of myocardial edema was traced and calculated from the T2w-STIR images by the use of a similar threshold-based approach (signal intensity >2 standard deviations of remote myocardium) [16]. Salvaged myocardium was quantified as the difference between the area of increased T2w-STIR signal (area at risk) and the area of LGE (IS), and myocardial salvage index (MSI) was calculated by normalizing MS for AAR as previously described [16–22]. All measurements were normalized to left ventricular (LV) mass. Reproducibility of CMR data in our laboratory were previously described. [2, 14, 21, 23]

### Statistical Analysis

Data were analyzed with SPSS software version 20.0 (SPSS Inc., Chicago, Illinois). Continuous variables were calculated as average values considering standard deviation, whereas categorical were calculated as percentages. Differences between means of continuous variables at different times to reperfusion were analyzed by 1-way analysis of variance by the use of a linear trend analysis; a post-hoc analysis with Bonferroni correction was made for differences between groups. The differences between categorical variables were analyzed with the chi-square test of Pearson. A Student *t* test for independent groups was used to assess differences in continuous variables between anterior versus non-anterior infarction, these tests were made without correction for multiple comparisons. Differences were considered statistically significant at a 2-sided p value <0.05. A multivariable logistic regression analysis was conducted considering as dependent variable myocardial salvage index after reperfusion. The median value of MSI detected in our study patients (= 0.41) was used to divide the whole population in two groups (MSI < 0.41 and MSI > 0.41). All variables presenting a significant value >0.25 at univariate analysis were included in the model. The stepwise method with backward elimination was used, and odds ratios (ORs) with 95% CIs were calculated. The model was evaluated with Hosmer and Lemeshow test.

## Results

### Clinical and Angiographic Data

The minimal dataset of this study may be found in *S1 Minidataset*. Clinical and angiographic data are summarized in Table 1. Coronary angioplasty was performed in left anterior descending artery (LAD) in 85 patients, in the right coronary artery in 83 patients, and in the left circumflex artery in 19 patients. Mean time from symptom onset to reperfusion was 152±149 min. No events suggesting reocclusion/stenosis were observed between PPCI and CMR examinations. For the purpose of the study, patients were subcategorized into 5 quintiles on the basis of the heart rate registered in the emergency room before the culprit lesion recanalization: <55 bpm (group I, n = 44), 55 to 64 bpm (group II, n = 35), 65 to 74 bpm (group III, n = 35), 75 to 84 bpm (group IV, n = 37), and ≥ 85 bpm (group V, n = 36). No differences on baseline clinical and angiographic characteristics were observed between groups, except for LAD infarcts that were more frequent in groups with higher heart rates (Table 1). In particular, no statistical differences between groups were observed in time to PCI, incidence of risk factors, prodromal angina, and TIMI score pre-PCI. The majority of patients showed TIMI flow 0–1 before coronary angioplasty. Concomitant treatment after reperfusion was similar between groups. (Table 1).

**Table 1:** Patients characteristics categorized by heart rate at hospital presentation.

Variable	Groups (n = 187 patients) Heart Rate					p values
	<55bpm (n = 44)	55-64bpm (n = 35)	65-74bpm (n = 35)	75–84 bpm (n = 37)	≥85bpm (n = 36)	
Age	56±11	61±9	62±11	58±10	57±10	0.054
Sex (male)	36 (81%)	32 (91%)	26 (74%)	33 (89%)	30 (83%)	0.309
Hypertension	17 (39%)	6 (17%)	15 (42%)	17 (45%)	16 (44%)	0.093
Diabetes	4 (9%)	2 (5.9%)	3 (8.6%)	6 (16%)	6 (16%)	0.503
Smoking	30 (69%)	17 (51%)	15 (45%)	23 (65%)	22 (61%)	0.362
Dislipidemia	24 (55%)	21 (61%)	21 (60%)	23 (62%)	17 (47%)	0.686
Family history of CAD	21(48%)	14 (41%)	16 (45%)	14 (37%)	11 (30%)	0.527
Prodromal Angina	4 (9%)	3 (8.6%)	5 (16%)	6 (16%)	3(8%)	0.146
Time to treatment (min)	147±131	124±122	119±130	176±142	161±149	0.106
Peak Troponin I (ng/ml)	83±112	78±73	120±109	142±147	89±116	0.081
LAD v/s no LAD	13 (29%)	9 (25%)	19 (54%)	23 (62%)	21 (58%)	0.001
TIMI pre-PCI 2/3	14 (32%)	9 (25%)	8 (23%)	7 (18%)	7 (19%)	0.088
Rentrop grade	0.23°0.70	0.25°0.45	0.39°0.33	0.20°0.71	0.32°0.78	0.488
ACE inhibitors/ARB	21 (87%)	31 (90%)	34 (97%)	30 (83%)	31 (84%)	0.412
Beta-blockers	34 (78%)	30 (85%)	29 (82%)	27 (73%)	29 (78%)	0.846
Statins	40 (91%)	30 (85%)	33 (94%)	32 (91%)	31 (84%)	0.636

Data are presented as median ± SD or number (%)

CAD = coronary artery disease; LAD = left anterior descending coronary artery; LCx = left circumflex coronary artery; MVO = microvascular obstruction; PCI = percutaneous coronary intervention; RCA = right coronary artery; TIMI = Thrombolysis In Myocardial Infarction; CC = collateral circulation; ACE = angiotensin-converting enzyme; ARB = angiotensin receptor blocker. Time to treatment = time form symptom onset to balloon inflation.

### Heart Rate and Infarct Size, Myocardial Edema, Microvascular Obstruction and Myocardial Salvage

**Fig 1:**
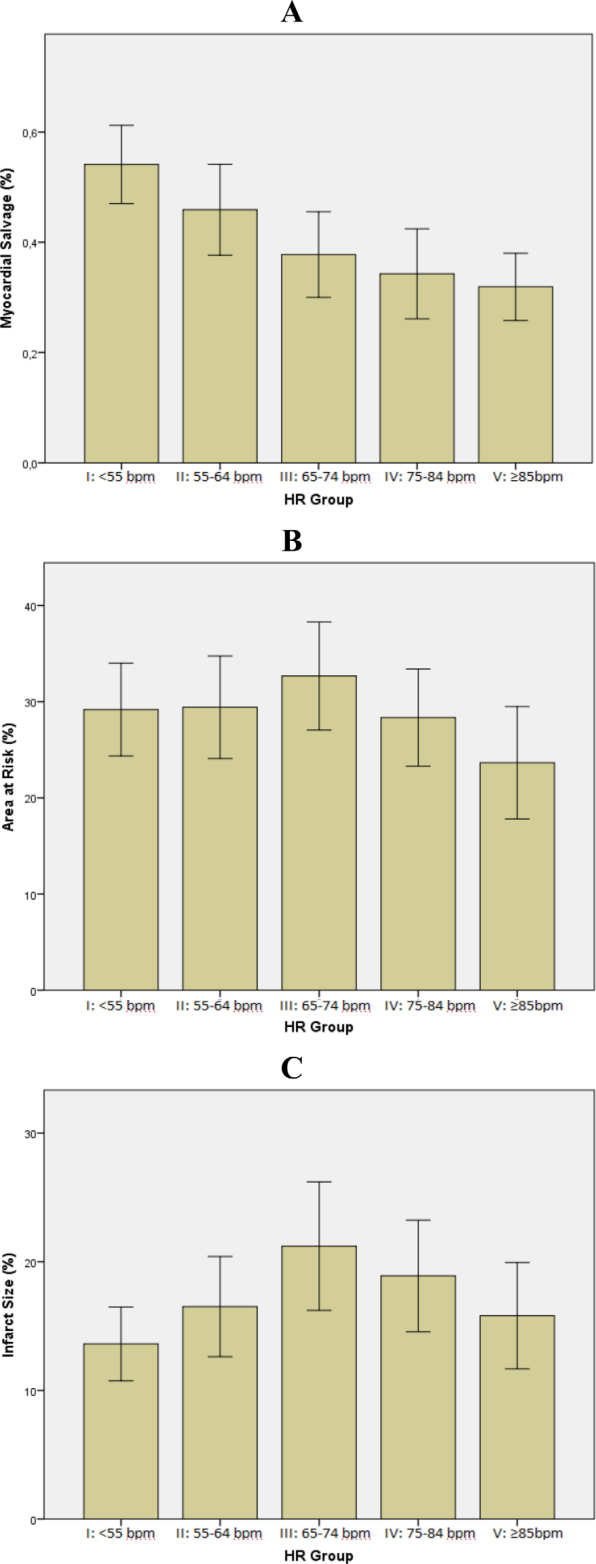
Bar graphs showing the influence of heart rate on myocardial salvage (A), area at risk (B) and infarct size (C) in patients categorized in quintiles according to heart rate at hospital admission (details in the text).

An infarcted region on LGE images was visualized in 180 out of 187 patients, corresponded to the infarct related artery territory distribution. Mean IS among groups was 17±12% of LV mass. Not significant changes of IS over heart rate were found (13%, 16%, 21%, 19% and 16%, respectively, p = 0.075) (Table 2; Fig 1). Increased signal intensity on T2w-STIR imaging (myocardial edema) was observed in all patients. The mean size of edema among groups was 28±16% of LV mass. In all patients, the location of T2w-STIR increased signal intensity corresponded to the territory of infarct related artery distribution. The extent of myocardial edema did not change significantly as heart rate increased (29%, 29%, 32%, 28% and 23%, respectively, p = 0.217). (Table 2; Fig 1). Also the presence of microvascular obstruction was similar between groups. Conversely, the MSI significantly reduced as heart rate increased (0.54, 0.46, 0.38, 0.34 and 0.32 respectively, p<0.001). In particular, a marked improvement in salvaged myocardium was observed as heart rate was ≤ 55 bpm (group I vs. III, p = 0.017; group I vs. IV, p = 0.001; group I vs. V, p<0.001), whereas a trend toward a larger amount of salvaged myocardium was observed between group II vs. V (p = 0.101), and no significant changes were observed between groups III, IV and V (Table 2; Fig 1). Dividing our study population into two groups according to HR median value, a higher MSI value was found for heart rate <70 bpm (0.49±0.23 vs 0.33±0.22; p<0.001), whereas AAR and IS showed no significant differences (30±16 vs 26±16; p = 0.124 and 16±11 vs 17±12; p = 0.333 respectively). At multivariable analysis heart rate [OR 0.95 (95%CI: 0.92–0.97) p = 0.001], peak troponin [OR 0.995 (95%CI: 0.991–0.998) p = 0.041] and the presence of MVO [OR 0.2 (95%CI: 0.075–0.537) p<0.001] were independently associated with myocardial salvage (Table 3).

**Table 2:** Cardiac magnetic resonance parameters after reperfusion categorized by heart rate at hospital presentation.

Variable	Groups (n = 187 patients) Heart Rate					p values
	<55bpm (n = 44)	55-64bpm (n = 35)	65-74bpm (n = 35)	75–84 bpm (n = 37)	≥85bpm (n = 36)	
AAR (%LV)	29±15	29±15	32±16	28±15	23±17	0.217
IS (%LV)	13±9	16±11	21±14	19±13	16±12	0.075
Presence of MVO	25 (56%)	22 (62%)	22 (62%)	16 (43%)	17 (47%)	0.316
MSI	0.54±0.22	0.46±0.21	0.38±0.20§	0.34±0.24†	0.32±0.17‡ •	<0.001
LVEF	51±7.2	50±8.1	48±8.3	47±9.2	47±8.9	0.082

Data are presented as median ± SD or number (%)

§ p = 0.017 vs. group I

†p = 0.001 vs. group I

‡ p<0.001 vs. group I

• p = 0.101 vs. group II

AAR = Area at Risk; IS = Infarct Size; LV = Left Ventricle; MVO = Microvascular obstruction; MSI = myocardial salvage index; LVEF = Left Ventricular Ejection Fraction

**Table 3:** Predictors of myocardial salvage in univariable and stepwise multivariable regression analysis.

Parameter	Univariable		Multivariable	
	P value	Odds Ratio (95%CI)	P value	Odds Ratio (95%CI)
SBP (mmHg)	0.218	0.991 (0.977–1)	-	-
DBP (mmHg)	0.047	0.977 (0.95–1)	-	-
Heart Rate (bpm)	<0.001	0.955 (0.937–0.974)	0.001	0.950 (0.920–0.970)
Peak Troponin	<0.001	0.993 (0.989–0.997)	0.041	0.995 (0.991–0.998)
CK MB	0.065	0.999 (0.997–1)	-	-
LAD/no LAD	NS	-	-	-
Age (years)	0.077	0.97 (0.94–1)	-	-
Sex (male)	0.200	1.68 (0.78–3.72)	-	-
Time to PCI	0.041	0.998 (0.996–1)	-	-
TIMI pre PCI	0.200	1.2 (0.9–1.6)	-	-
TIMI post PCI	0.147	2.1 (0.77–5.73)	-	-
Hypertension	NS	-	-	-
Diabetes	NS	-	-	-
Hyperlipemia	NS	-	-	-
Smoke habit	NS	-	-	-
Angina	NS	-	-	-
Family history of CAD	NS	-	-	-
MVO presence	<0.001	0.341 (0.188–0.619)	<0.001	0.2 (0.075–0.537)

SBP = Systolic Blood Pressure; DBP = Diastolic Blood Pressure; CK MB = Creatine Kinase Myocardial Band; LAD = Left Anterior Descending Coronary Artery; TIMI = Thrombolysis In Myocardial Infarction; PCI = Percutaneous Coronary Angioplasty; CAD = Coronary Artery Disease MVO = Microvascular Obstruction

### Heart Rate and Infarct Location

The relation between infarct location and heart rate was depicted in *Table 4.* LAD infarcts were associated to higher heart rates and they were more frequently found in groups III, IV and V (29%, 25%, 54%, 62% and 58% respectively, p = 0.001). Notably, when LAD was occluded a higher amount of edematous (32±17 vs. 25±14, p = 0.002) and necrotic (19±13 vs. 15±10, p = 0.015) myocardium was found. On the opposite, MSI did not significantly change in relation the occlusion site (0.40±0.23 vs. 0.42±0.25, p = 0.672). (Table 4).

**Table 4:** Average values of cardiac magnetic resonance parameters in anterior vs. non-anterior infarction.

Variable	Groups (n = 187 patients)		P values
	LAD (n = 85)	No-LAD (n = 102)	
**Area at Risk (%LV)**	32±17	25±14	0.002
**Infarct Size (%LV)**	19±13	15±11	0.015
**Myocardial Salvage Index**	0.40±0.21	0.42±0.24	0.672
**LVEF**	47±8.8	50±8.7	0.018

Abbreviations as in previous tables

## Discussion

In the present study, we described, for the first time in human beings, benefits in terms of myocardial salvage associated with a lower heart rate in patients with successfully reperfused STEMI. Noninvasive myocardial tissue characterization provided by CMR enabled us to differentiate reversible and irreversible myocardial injury (myocardium at risk and myocardial infarction, respectively) and consequently to determine the presence and extent of salvaged myocardium. Major findings of this study are as follows: 1) Progressive reduction in myocardial salvage is present as heart rate increases during the occlusion-phase 2) Site of the lesion is associated with the extent of the area at risk and infarct size but not related to myocardial salvage. These results were observed irrespective of any other known factors influencing myocardial salvage, such us time to reperfusion [22], presence of microvascular obstruction, infarct size, gender [14], pre-infarction angina [23] and TIMI score pre-PCI [24].

### Heart Rate and Necrotic Wave Progression

Other than defining the “*wavefront phenomenon”*, Reimer and Jennings [1] were also able to describe how it could be stopped by reperfusion and influenced in its progression by other factors [25]. Maroko et al. [4] observed that when heart rate rise-up after pacing or isoproterenol, during LAD occlusion, a higher ST-segment elevation was present. Moreover Przyklenk et al. [5] demonstrated, during experimental coronary occlusion, higher percentage of area at risk becoming necrotic as heart rate increases. Heart rate seemed to be able to influence the progression of the necrotic wave. It was hypothesized that a reduction of diastolic time at high heart rates may reduce blood flow from collateral vessels and increase oxygen consumption. Both factors leaded to a less amount of salvaged myocardium [5]. On this rationale, several studies were carried out testing drugs being able to reduce heart rate in order to slow down the spreading of the necrosis. Reimer and Jennings [26] were also able to achieve an improvement in myocardium salvage through the administration of propranolol in dogs after circumflex coronary artery ligation. More than three decades later the paradigm “slow the heart rate to preserve myocardium viability” has remained valid, as showed by recent studies in which the administration of drugs able to reduce heart rate and oxygen consumption were able to improve myocardial salvage in animal models of reperfused myocardial infarction [6, 27]. Heart rate reduction by Ivabradine appears to have a positive effect on coronary collateral function in patients with chronic stable coronary artery disease [28]. Taken all together these observations suggest that higher heart rate is also a cause, rather than only a consequence, of a wider myocardial injury during the coronary artery occlusion.

Coherently with observations in animals, in our study we found a constant reduction of myocardial salvage as heart rate, registered at the admission before reperfusion, increased. At multivariate analysis, even when correcting for every known factors influencing myocardial salvage, heart rate remains an independent predictor of myocardial salvage. The result reinforces the hypothesis that high heart rate is able to accelerate the progression of the necrotic wave, facilitating necrosis in ischemic but still viable cells, by exposing them to a higher metabolic demand and to a lower perfusion through collateral circulation. We found no significant changes in IS between the groups. On the other hand, the final infarct extent is influenced by several factors other than the spreading of necrosis within the area at risk. In particular, the extent of the bed of perfusion of the culprit vessel is one of its major determinants, since AAR accounts for 70% of the variability in the extent of myocardial necrosis [25]. Although not statistically significant, variability in AAR extent between groups was present, due to a significant difference in anterior infarcts frequency. These factors could be important confounders when taking into account infarct size, but less important when measuring myocardial salvage. In our patients, and in accordance with our previous report [29], MSI was independent of culprit lesion site. On the opposite, AAR and IS largely vary in relation to the site of the lesion; consequently MSI can be considered a better index of the progression of the necrotic wave and a better surrogate end-point in testing novel reperfusion strategies, as already proposed[2, 3].

For the first time CMR allowed us to non-invasively verify in humans these results by measurement of both AAR and IS in vivo, and from their difference the amount of the salvaged myocardium. Notably, these measurements can be obtained in a single exam performed between one to five days after the infarction.

### Clinical Implications

Heart rate has been considered for years as an important prognostic factor, being able to predict both all-cause-mortality and cardiovascular mortality [30], either in patients with stable coronary artery disease and left ventricular dysfunction [31] or with heart failure [32]. In patients with acute coronary syndrome, heart rate at discharge has been found to be related to the mortality at follow-up [33, 34]. Moreover, Parodi et al. [35] observed that heart rate at presentation ≥80 bpm, in patients with STEMI undergoing primary PCI, was able to predict mortality at a 6 months follow-up. Despite these evidences, it is not yet demonstrated whether in the context of acute coronary syndrome, heart rate can be also considered as a risk factor other than a marker of risk. In our study, we observed for the first time in humans how higher heart rates were related to wider injuries in the myocardium, linking what observed in animals at the bench of the laboratory with the poor clinical outcomes observed at the bedside. Though recently introduced, MSI has been proved to be an important prognostic marker, being able to predict not only mortality but also early ST-segment normalization and left ventricular remodeling in patients with reperfused STEMI: a reduction in salvaged myocardium is related to a worsening of prognosis [2, 3]. Leading to a reduction in myocardial salvage, higher heart rate could partially explain the higher mortality rate observed. Moreover, it could be proposed that lowering heart rate during the progression of the necrotic wave could be useful to preserve myocardium viability within the AAR, and consequently improve prognosis. On these basis a trial was recently conducted [36], administrating Ivabradine in patients with STEMI undergoing PPCI. Safety and tolerability were positively assessed, but it failed to show a significant reduction in IS. However, the drug was administered after reperfusion, probably minimizing possible benefits. Moreover, the amount of salvaged myocardium was not measured.

### Study Limitations

Even though the number of patients included in this study is quite large, our preliminary data needed to be confirmed by larger multicenter trails aiming to definitively demonstrate the independent role of heart rate on myocardial salvage extent. In particular, the absence of significant differences in MVO extent between groups should be confirmed in a larger population. Time to reperfusion is a stronger determinant of myocardial salvage and of the final extent of infarct size, however in a selected population treated according to the current guidelines (mean time from symptom onset to reperfusion was 152±149 min in our study population), also the basal heart rate has a strong importance. CMR studies were performed from day 4 to 5 after the infarction. A recent experimental study [37] showed the amount of edema may be variable soon after myocardial infarction as edematous reaction almost disappears 24h after reperfusion and progressively reappears till a maximum on day 7. However, by the study design the authors did not correlate the area at risk with the extension of CMR-visualized edema, thus any conclusions in this regard are speculative. Conversely several clinical and experimental studies [2, 3,7, 8, 16–19, 21–24, 38] strongly support the hypothesis that water content, as assessed by and T2w-STIR sequences, in the first week after acute myocardial infarction reflects the area at risk in humans. Future studies will need to better address this point. Unstable patients (Killip class III-IV) and unsuccessful revascularized patients were excluded from this study, therefore the results of this paper may not be extended to the general population with acute myocardial infarction. Finally, in this study CMR was performed as part of a research protocol and did not contribute to the care of patients.

## Conclusion

In the present study we observed that high heart rates registered before performing coronary angioplasty in timely reperfused patients with STEMI are associated with a reduction in salvaged myocardium. In particular, salvaged myocardium significantly reduced when heart rate at presentation is ≥85 bpm. Possible benefits in therapy able to reduce heart rate in such a clinical setting should be hypothesized.

## Supporting Information

S1 Minidataset (XLSX)

## References

[1] Reimer KA, Lowe JE, Rasmussen MM, Jennings RB. The wavefront phenomenon of ischemic cell death. 1. Myocardial infarct size vs. duration of coronary occlusion in dogs. Circulation 1977;56:786–94. 912839 10.1161/01.cir.56.5.786

[2] Masci PG, Ganame J, Strata E, Desmet W, Aquaro GD, Dymarkowski S, et al Myocardial salvage by CMR correlates with LV remodeling and early ST-segment resolution in acute myocardial infarction. J Am Coll Card Cardiovasc Imaging 2010;3:45–51. 10.1016/j.jcmg.2009.06.016 20129530

[3] Eitel I, Desch S, de Waha S, Fuernau G, Gutberlet M, Schuler G, et al Long-term prognostic value of myocardial salvage assessed by cardiovascular magnetic resonance in acute reperfused myocardial infarction. Heart 2011;97:2038–45. 10.1136/heartjnl-2011-300098 21990384

[4] Maroko PR, Kjekshus JK, Sobel BE, Watanabe T, Covell JW, Ross J Jr, et al Factors Influencing Infarct Size Following Experimental Coronary Artery Occlusions. Circulation 1971;43:67–82. 5540853 10.1161/01.cir.43.1.67

[5] Przyklenk K, Vivaldi MT, Schoen FJ, Malcolm J, Arnold O, Kloner RA. Salvage of Ischaemic myocardium by reperfusion: importance of collateral blood flow and myocardial oxygen demand during occlusion. Cardiovascular Research 1986;20:403–414. 3779738 10.1093/cvr/20.6.403

[6] Larsen JR, Sivesgaard K, Christensen SD, Hønge JL, Hasenkam JM. Heart rate limitation and cardiac unloading in sevoflurane postconditioning. Acta Anaesthesiol Scand 2012;56:57–65. 10.1111/j.1399-6576.2011.02580.x 22103708

[7] Friedrich MG1, Abdel-Aty H, Taylor A, Schulz-Menger J, Messroghli D, Dietz R. The salvaged area at risk in reperfused acute myocardial infarction as visualized by cardiovascular magnetic resonance. J Am Coll Cardiol 2008;51:1581–7. 10.1016/j.jacc.2008.01.019 18420102

[8] Eitel I, Desch S, Fuernau G, Hildebrand L, Gutberlet M, Schuler G, et al Prognostic significance and determinants of myocardial salvage assessed by cardiovascular magnetic resonance in acute reperfused myocardial infarction. J Am Coll Cardiol 2010;55:2470–9. 10.1016/j.jacc.2010.01.049 20510214

[9] Agati L, Voci P, Bilotta F, Luongo R, Autore C, Penco M, et al Influence of residual perfusion within the infarct zone on the natural history of left ventricular dysfunction after acute myocardial infarction. J Am Coll Cardiol 1994;24:336–42. 8034865 10.1016/0735-1097(94)90285-2

[10] Galiuto L, Garramone B, Scarà A, Rebuzzi AG, Crea F, La Torre G, et al The extent of microvascular damage at myocardial contrast echocardiography is superior to other known indexes of post-infarct reperfusion in predicting left ventricular remodeling. Results of the multicenter study “Acute Myocardial Infarction Contrast Imaging” (AMICI). J Am Coll Cardiol 2008;51:552–9. 10.1016/j.jacc.2007.09.051 18237684

[11] Gerber BL. Risk area, infarct size, and the exposure of the wavefront phenomenon of myocardial necrosis in humans. Eur Heart J 2007;28:1670–2. 17586810 10.1093/eurheartj/ehm213

[12] Rentrop KP, Cohen M, Blanke H, Philips RA. Changes in collateral channel filling immediately after controlled coronary artery occlusion by an angioplasty balloon in human subjects. J Am Coll Cardiol 1985;5:587–92. 3156171 10.1016/s0735-1097(85)80380-6

[13] Simonetti OP, Finn JP, White RD, Laub G, Henry DA. “Black blood” T2-weighted inversion-recovery MR imaging of the heart. Radiology 1996;199:49–57. 8633172 10.1148/radiology.199.1.8633172

[14] Canali E, Masci P, Bogaert J, Bucciarelli Ducci C, Francone M, McAlindon E, et al Impact of gender differences on myocardial salvage and post-ischemic left ventricular remodeling after primary coronary angioplasty: new insights from cardiovascular magnetic resonance. Eur Heart J Cardiovasc Imaging 2012;13:948–53. 10.1093/ehjci/jes087 22531464

[15] Bondarenko O, Beek AM, Hofman MB, Kühl HP, Twisk JW, van Dockum WG, et al Standardizing the definition of hyperenhancement in the quantitative assessment of infarct size and myocardial viability using delayed contrast-enhanced CMR. J Cardiovasc Magn Reson 2005;7:481–5. 15881532 10.1081/jcmr-200053623

[16] Abdel-Aty H1, Zagrosek A, Schulz-Menger J, Taylor AJ, Messroghli D, Kumar A, et al Delayed enhancement and T2-weighted cardiovascular magnetic resonance imaging differentiate acute from chronic myocardial infarction. Circulation 2004;109:2411–6. 15123531 10.1161/01.CIR.0000127428.10985.C6

[17] Aletras AH, Tilak GS, Natanzon A, Hsu LY, Gonzalez FM, Hoyt RF Jr, et al Retrospective determination of the area at risk for reperfused acute myocardial infarction with T2-weighted cardiac magnetic resonance imaging: histopathological and displacement encoding with stimulated echoes (DENSE) functional validations. Circulation 2006;113:1865–70. 16606793 10.1161/CIRCULATIONAHA.105.576025

[18] Abdel-Aty H, Simonetti O, Friedrich MG. T2-weighted cardiovascular magnetic resonance imaging. J Magn Reson Imaging 2007;26: 452–9. 17729358 10.1002/jmri.21028

[19] Bøtker HE, Kaltoft AK, Pedersen SF, Kim WY. Measuring myocardial salvage. Cardiovascular Research 2012;94: 266–275. 10.1093/cvr/cvs081 22311720

[20] Stork A, Lund GK, Muellerleile K, Bansmann PM, Nolte-Ernsting C, Kemper J, et al Characterization of the peri-infarction zone using T2-weighted MRI and delayed enhancement MRI in patients with acute myocardial infarction. Eur Radiol 2006;16:2350–7. 16625349 10.1007/s00330-006-0232-3

[21] Dymarkowski S, Ni Y, Miao Y, Bogaert J, Rademakers F, Bosmans H, Marchal G. Value of T2-weighted magnetic resonance imaging early after myocardial infarction in dogs: comparison with bis-gadolinium-mesoporphyrin enhanced T1-weighted magnetic resonance imaging and functional data from cine magnetic resonance imaging. Invest Radiol 2002;37:77–85. 11799331 10.1097/00004424-200202000-00005

[22] Francone M, Bucciarelli-Ducci C, Carbone I, Canali E, Scardala R, Calabrese FA, et al Impact of time to reperfusion on myocardial salvage, infarct size and microvascular damage in patients undergoing primary coronary angioplasty for ST-elevation myocardial infarction: insight from cardiovascular magnetic resonance. J Am Coll Cardiol 2009;54:2145–2153. 10.1016/j.jacc.2009.08.024 19942086

[23] Masci PG, Andreini D, Francone M, Bertella E, De Luca L, Coceani M, et al Prodromal angina is associated with myocardial salvage in acute ST-segment elevation myocardial infarction Eur Heart J Cardiovasc Imaging 2013;14:1041–8. 10.1093/ehjci/jet063 23793878

[24] Ortiz-Pérez JT, Lee DC, Meyers SN, Davidson CJ, Bonow RO, Wu E. Determinants of myocardial salvage during acute myocardial infarction: evaluation with a combined angiographic and CMR myocardial salvage index. J Am Coll Cardiol Cardiovasc Imaging 2010;3:491–500. 10.1016/j.jcmg.2010.02.004 20466344

[25] Reimer KA, Jennings RB, Cobb FR, Murdock RH, Greenfield JC Jr, Becker LC, et al Animal models for protecting ischemic myocardium: results of the NHLBI Cooperative Study. Comparison of unconscious and conscious dog models. Circ Res 1985;56:651–5. 3838923 10.1161/01.res.56.5.651

[26] Rasmussen MM, Reimer KA, Kloner RA, Jennings RB. Infarct Size Reduction by Propranolol before and after Coronary Ligation in Dogs. Circulation 1977;56:795–804. 10.1161/01.cir.56.5.794 912840

[27] Heusch G1, Skyschally A, Gres P, van Caster P, Schilawa D, Schulz R. Improvement of regional myocardial blood flow and function and reduction of infarct size with Ivabradine: protection beyond heart rate reduction. Eur Heart J 2008; 29: 2265–2275. 10.1093/eurheartj/ehn337 18621770

[28] Gloekler S1, Traupe T, Stoller M, Schild D, Steck H, Khattab A, et al The effect of heart rate reduction by ivabradine on collateral function in patients chronic stable coronary artery disease. Heart 2013;0:1–7. 10.1136/heartjnl-2013-304880 24186565

[29] Masci PG, Ganame J, Francone M, Desmet W, Lorenzoni V, Iacucci I, et al Relationship between location and size of myocardial infarction and their reciprocal influences on post-infarction left ventricular remodelling. Eur Heart J. 2011;32:1640–8. 10.1093/eurheartj/ehr064 21398642

[30] Kannel WB, Kannel C, Paffenbarger RS Jr, Cupples LA. Heart rate and cardiovascular mortality: the Framingham Study. Am Heart J. 1987;113:1489–94. 3591616 10.1016/0002-8703(87)90666-1

[31] Fox K, Ford I, Steg PG, Tendera M, Ferrari R. Ivabradine for patients with stable coronary artery disease and left ventricular systolic dysfunction (BEAUTIFUL): a randomized, double blind, placebo-controlled trial. Lancet 2008; 372: 807–16. 10.1016/S0140-6736(08)61170-8 18757088

[32] Swedberg K, Komajda M, Böhm M, Borer JS, Ford I, Dubost-Brama A, et al Ivabradine and outcomes in chronic heart failure (SHIFT): a randomized placebo-controlled study. Lancet 2012;11;376:875–8.

[33] Zuanetti G, Mantini L, Hernández-Bernal F, Barlera S, di Gregorio D, et al Relevance of heart rate as a prognostic factor in patients with acute myocardial infarction: insights from the GISSI-2 study. Eur Heart J 1998: F19–F26. 9651731

[34] Antoni ML, Boden H, Delgado V, Boersma E, Fox K, Schalij MJ, et al Relationship between discharge heart rate and mortality in patients after acute myocardial infarction treated with primary percutaneous coronary intervention. Eur Heart J 2012; 33, 96–102. 10.1093/eurheartj/ehr293 21862462

[35] Parodi G, Bellandi B, Valenti R, Memisha G, Giuliani G, Velluzzi S, et al Heart rate as an independent prognostic risk factor in patients with acute myocardial infarction undergoing primary percutaneous coronary intervention. Atherosclerosis 2010;211: 255–259. 10.1016/j.atherosclerosis.2010.02.017 20226462

[36] Steg PG, Lopez-de-Sà E, Schiele F, Hamon M, Meinertz T, Goicolea J, et al Safety of intravenous ivabradine in acute ST-segment elevation myocardial infarction patients treated with primary percutaneous coronary intervention: a randomized, placebo-controlled, double-blind, pilot study. Eur Heart J Acute Cardiovasc Care 2013;2: 270–9. 10.1177/2048872613489305 24222839 PMC3821820

[37] Fernández-Jiménez R, Sánchez-González J, Agüero J, García-Prieto J, López-Martín GJ, García-Ruiz JM, et al Myocardial edema after ischemia/reperfusion is not stable and follows a bimodal pattern. J Am Coll Cardiol 2015; 65:315–23. 10.1016/j.jacc.2014.11.004 25460833

[38] Berry C, Kellman P, Mancini C, Chen MY, Bandettini WP, Lowrey T, et al Magnetic resonance imaging delineates the ischemic area at risk and myocardial salvage in patients with acute myocardial infarction. Circ Cardiovasc Imaging. 2010 3:527–35. 10.1161/CIRCIMAGING.109.900761 20631034 PMC2966468

